# Neddylation and anti-tumor immunity

**DOI:** 10.18632/oncotarget.28019

**Published:** 2021-10-12

**Authors:** Xiaoguang Wang, Scott Best, Alexey V. Danilov

**Affiliations:** ^1^Department of Hematology and Hematopoietic Stem Cell Transplant, City of Hope National Medical Center, Duarte, CA, USA; ^2^Molecular and Cellular Biology, University of Washington, Seattle, WA, USA

**Keywords:** neddylation, T cell, pevonedistat

## Abstract

Contrary to chemotherapy, novel targeted therapies are associated with diverse immunomodulatory effects. Nedd8 is a small ubiquitin-like modifier that is involved in regulation of protein degradation. Neddylation is a promising target in cancer. Pevonedistat, a small molecule inhibitor of the Nedd8-activating enzyme, demonstrates pre-clinical activity in multiple tumor types. Recent studies have revealed that neddylation is important in immunity. We and others have shown that interfering with neddylation causes downstream immunomodulatory effects potentially leading to enhanced anti-tumor immunity. Thus, Nedd8 is a promising target in immuno-oncology.

The ubiquitin-proteasome system (UPS) is a common pathway which controls degradation of unwanted or misfolded proteins in cells. Proteins targeted for degradation are labeled with ubiquitin chains by E3 ubiquitin ligases [1]. The Cullin-RING E3 ubiquitin ligases (CRLs) represent the largest family of E3 ligases, which contribute approximately 20% of UPS-mediated protein degradation [2]. Targeting protein degradation pathway has precedence in the clinic. Bortezomib, carfilzomib and ixazomib are proteasome inhibitors which received regulatory approvals in the therapy of multiple myeloma. Lenalidomide and pomalidomide modulate Cereblon, part of an E3 ligase complex, resulting in increased secretion of IL-2 by T cells, a shift towards the Th1 phenotype and improved immune synapse formation [3]. Moreover, lenalidomide was shown to enhance the function of chimeric antigen receptor T cells [4].

The conjugation of the ubiquitin-like modifier NEDD8 to Cullins (neddylation) is essential for the conformational change and subsequent ubiquitination of CRL (Cullin RING ligase) substrates [5]. As a post-translational modification process, neddylation is a three-step sequential enzymatic reaction: 1. NEDD8 activation by the E1 NEDD8-activating enzyme (NAE, NAE1/UBA3 heterodimer) in an ATP-dependent manner; 2. NEDD8 transfer to the E2 NEDD8-conjugating enzymes (UBC12 and UBE2F) via a thioester linkage; 3. NEDD8 conjugation to cullin activates the ubiquitin ligase activity of the CRL [6, 7]. Pevonedistat is a small molecule which covalently adducts with NEDD8, leading to effective inhibition of NAE via competitive binding. Consequently, NEDD8 is prevented from conjugation to CRLs, thus leading to CRL deactivation and accumulation of CRL-dependent substrates [8]. As a first-in-class NAE inhibitor, pevonedistat demonstrated pre-clinical and early clinical efficacy across multiple tumor types [9–14]. Furthermore, we and others have reported additive cytotoxic activity of NAE inhibition in combination with chemotherapeutic and targeted agents [15–20]. Pevonedistat has entered clinical trials in solid tumors and hematologic malignancies. Our group is conducting a Phase I clinical trial investigating pevonedistat in non-Hodgkin lymphoma (NCT03479268).

Recent reports have implicated neddylation in regulation of immune cell function, including proliferation, maturation, effector cell function and signal transduction [21–25]. Meanwhile, pharmacologic and genetic manipulation of the neddylation pathway has been shown to modulate T-cell mediated immune responses [26]. A significant body of literature suggests that neddylation regulates T-cell functionality. In a pivotal study, genetic knockdown of the Nedd8-conjugating enzyme Ubc12 in murine CD4^+^ T cells led to diminished proliferation, skewed Th1/Th2 differentiation and reduced cytokine production [21]. *In vivo* treatment with pevonedistat was shown to have comparable effects in a malaria murine model, where UBA3 deficiency significantly compromised survival, activation, and proliferation of CD4 T cells and impaired Th1/Tfh differentiation [22]. Similarly, downmodulation of sensitive to apoptosis gene (SAG), regulator of Cullin neddylation, reduced T-cell activation, proliferation, and cytokine release in murine T cells [23]. Recently, our group has shown that *in vitro* exposure to pevonedistat downregulated activation of proximal T-cell receptor (TCR) signaling, accompanied by suppression of NF-κB-regulated genes and IL-2 signaling in T cells derived from patients with chronic lymphocytic leukemia (CLL) [24]. However, we also found that targeting neddylation may exert immune modulation that ultimately leads to enhanced anti-tumor effects. Specifically, we demonstrated that NAE inhibition with pevonedistat suppressed induction of FoxP3-positive CD4 Tregs in *in vitro* conditions. This result was confirmed in immunized mice [24]. Furthermore, we have observed increased T-cell polarization towards Th1 phenotype *in vitro* and *in vivo*, accompanied by an increase of IFNγ production. Our results are partially supported by Friend et al who observed that CRLs suppress TCR signaling and IL-2 production in murine T-cell hybridomas, a phenomenon reversed by pevonedistat [25].

Although pevonedistat may suppress the ability of dendritic cells to stimulate murine and human allogeneic T-cell responses [27], treatment with pevonedistat was shown to significantly inhibit infiltration of immune suppressor cells, including tumor-associated macrophages (TAMs) and myeloid derived suppressor cells (MDSCs), and promoted CD8 T cell infiltration in lung cancer models [28].

Immune checkpoint (e.g., PD-1, CTLA-4) has become an important target in cancer. There is paucity of data as to how neddylation and other UPS components regulate expression, turnover and function of these molecules. We have found that targeting NAE with pevonedistat significantly enhanced CTLA-4 expression in human T cells [24]. The mechanism for this is poorly understood but may depend on NAE-mediated control of several key signaling pathways. CTLA-4 is positively regulated by the transcription factor GATA3 [29]. Proteasome inhibition was shown to induce GATA3, upregulate CTLA-4 expression and thereby efficiently suppress the proliferation of CD4 T cells *in vitro* [29]. A recent study found that SKP1-Cul1-F-box E3 ligase complex induces degradation of GATA3 in a GSK3-dependent manner [30], and targeting this complex may therefore lead to enhanced expression of CTLA-4. On the other hand, our data shows that pevonedistat markedly increases HIF-1α expression in CD3/28-stimulated T cells [24], a finding consistent with the fact that ubiquitination of HIF-1α is mediated by VHL–Cul2-EloBC E3 ligase. HIF-1α is critical to metabolic transition to glycolysis, a process indispensable in T-cell proliferation and effector functions [31]. Loss of HIF-1α in CD8 T cells was shown to reduce activation, tumor infiltration and tumor cell killing, and alter tumor vascularization [32]. By contrast, VHL-deficient cytotoxic T lymphocytes displayed enhanced control of persistent viral infection and neoplastic growth via HIFs [33]. Checkpoint receptors, including TIM3, PD-1, CTLA-4 and LAG-3, were also downregulated in CD8 T cells deficient in HIF-1α, and thus it is possible that enhanced CTLA-4 expression observed by us may be in part mediated by NAE interference with HIF-1α signaling pathway [32].

Genetic depletion of key neddylation pathway enzymes or pevonedistat treatment was also shown to markedly elevate PD-L1 expression in gliomas, in part due to a block in degradation of MYC protein [18, 34]. Furthermore, inactivation of cullin 3–SPOP E3 ligases blocked degradation of PD-L1, causing increased PD-L1 levels and reduced numbers of tumor-infiltrating lymphocytes in mouse tumors and in primary human prostate cancer specimens [35]. While PD-L1 induction attenuated T cell-mediated killing in this model, concurrent targeting of NAE and PD-L1 restored anti-tumor immunity. Targeting NAE may also enhance tumor antigenicity. Thus, cancer cells exhibiting enhanced microsatellite instability (MSI) were dependent on neddylation to clear misfolded protein aggregates resulting from destabilizing mutations [17]. In these cells, treatment with pevonedistat induced immunogenic cell death when PD-1 was blocked. These studies pave the way for rationally designed therapeutic strategies employing a neddylation inhibitor and a checkpoint inhibitor. The effects of concurrent targeting neddylation and CTLA-4 and other immune checkpoint molecules have not been investigated thus far.

In sum, a significant body of literature highlights direct anti-tumor effects of pevonedistat, firmly establishing neddylation as a therapeutic target in cancer. Provocative new data uncover a hitherto poorly understood immunomodulatory function of neddylation, with a potential to redefine its place in immuno-oncology. Such advances are highly relevant in the current age of cancer therapeutics, as they open avenues to enhance efficacy of existing therapies as well as develop novel mechanistic approaches to boost anti-tumor immunity.
